# Factors Associated With Surgical Site Infection Among Patients Undergone Abdominal Surgery in Northwestern Ethiopia: A Retrospective Cross‐Sectional Study

**DOI:** 10.1002/hsr2.71095

**Published:** 2025-07-21

**Authors:** Tesfaye Shumet Mekonnen, Shegaw Getinet, Amare Mebrat Delie, Eneyew Talie Fenta, Fassikaw Kebede Bizuneh, Wubetu Woyraw

**Affiliations:** ^1^ Department of Epidemiology and Biostatics, School of Public Health Debre Markos University Debre Markos Amhara Region Ethiopia; ^2^ Department of Surgery, School of Medicine Bahir Dar University Bahir Dar Amhara Region Ethiopia; ^3^ Department of Public Health, College of Health Science Injibara University Injibara Ethiopia; ^4^ Department of Nutrition, College of Health Science Debre Markos University Debre Markos Ethiopia

**Keywords:** abdominal surgery, associated factors, Ethiopia, surgical site infection

## Abstract

**Background and Aim:**

Among the myriad surgical procedures, abdominal surgeries stand out for their increased susceptibility to surgical site infections, owing to the intricate nature of the abdominal cavity. This study aimed to evaluate the prevalence and factors associated with surgical site infections among patients who underwent abdominal operations at Bichena Hospital.

**Methods:**

A retrospective cross‐sectional study conducted at a hospital involved 164 patients who underwent abdominal operations in the last 2 years. The prevalence of surgical site infection was estimated. Binary Logistic regression analysis was conducted and a significance level of *p*‐value ≤ 0.05 was adopted to identify statistically significant factors influencing surgical site infection following open abdominal surgery.

**Results:**

The occurrence of surgical site infection was determined to be 26.8% (95% CI: 20.1%, 33.5%). Patients with concurrent medical conditions were over three times more likely (AOR = 3.37) to develop SSI compared to those without such conditions. Regarding hospital length of stay, patients with shorter stays had a significantly lower likelihood of developing SSI. Specifically, those with stays of 5–7 days had a 91% lower likelihood of SSI (AOR = 0.09), those with 8–14 days had a 78% lower likelihood (AOR = 0.22), and those with stays of 15–21 days had a 72.4% lower likelihood (AOR = 0.28), compared to patients with a hospital stay of 22 days or longer. The 22‐day cutoff was determined based on the distribution of hospital stays within the study population, where longer stays beyond this period were associated with a higher incidence of infections.

**Conclusion:**

The study found a high incidence of SSIs at the institution. The presence of concurrent medical conditions and extended hospital stays was identified as a significant factor contributing to the occurrence of SSIs.

**Abbreviations**
AOR: adjusted odds ratio
AUROC: area under the receiver operating characteristics
CDC: Centers for Disease Control and Prevention
CI: confidence interval
COR: Crude odd ratio
EC: Ethiopian calendar
MRN: medical record number
NINSS: Nosocomial Infection National Surveillance Scheme
OR: odds ratio
SSI: surgical site infection
WHO: World Health Organization

## 1 Background

A Surgical Site Infection (SSI) is characterized by the proliferation of pathogenic microorganisms at an incision site, either within the skin and subcutaneous fat (superficial) or in deeper layers, or in an organ or cavity if exposed during surgery [1]. SSIs represent a formidable challenge in healthcare systems worldwide, imposing significant clinical and economic burdens [2]. It ranks among the most prevalent surgical complications, constituting 38% of all hospital‐acquired infections globally [3, 4, 5]. SSIs contribute to a staggering 500,000 infections and 3.7 million excess hospital days annually in the United States alone [6]. Particularly after major abdominal surgeries, SSIs can affect 2.5% to 10% of patients in high‐income countries, with rates varying based on the quality of healthcare infrastructure and adherence to prevention protocols [7, 8, 9]. However, in sub‐Saharan Africa, including Ethiopia, the rates are notably higher, ranging up to 30.9% [7].

Among the diverse range of surgical procedures, abdominal surgeries are particularly prone to the development of SSIs due to the complex nature of the abdominal cavity and its vulnerability to microbial contamination [9, 10]. In Ethiopia, given the distinctive challenges and diverse patient populations in the healthcare landscape, it is crucial to comprehend the prevalence and associated factors of surgical site infections (SSIs) after abdominal surgery [8, 11]. This understanding is essential for enhancing patient care and optimizing surgical outcomes.

Numerous risk factors identified in various literature sources contribute to the predisposition to SSI, including pre‐existing illnesses, duration of the operation, length of hospital stay, wound class, and wound contamination [12, 13, 14]. Recent studies challenge the long‐held belief that surgical technique is the primary determinant of SSIs, emphasizing instead the importance of systemic factors such as age, gender, lifestyle, and the presence of comorbidities [3, 15, 16, 17]. Factors like the surgical site, incision size and depth, antibiotic prophylaxis, instruments and suture materials, wound closure techniques, and patient‐related factors like co‐morbidities and lifestyle habits such as smoking significantly influence the occurrence of these events [17, 18, 19].

Ethiopia, a country with a population exceeding 118 million people, grapples with a healthcare system that faces multifaceted challenges, including limited resources, uneven healthcare infrastructure, and varying healthcare practices across regions [20, 21]. Despite the recognized global impact of SSIs on patient outcomes and healthcare costs, specific insights into the magnitude and determinants of SSIs following abdominal surgery within the Ethiopian context are notably lacking [22, 23, 24].

This study aims to bridge this knowledge gap by conducting a thorough investigation into the prevalence and associated factors of SSIs among patients who have undergone abdominal surgery in Ethiopia. The findings of this study will not only contribute to the global understanding of SSI dynamics but will also provide valuable insights tailored to the Ethiopian healthcare context [25]. By identifying specific factors associated with SSIs in this population, healthcare practitioners and policymakers can develop targeted interventions to minimize the occurrence of these infections, enhance patient safety, and optimize the overall quality of surgical care in Ethiopia [15]. This study also aims to inform the development of actionable, evidence‐based strategies for SSI prevention in low‐resource settings. By identifying key risk factors such as co‐morbidities, hospital stay duration, and surgical practices, the findings will guide the creation of targeted interventions for SSI prevention, including optimized preoperative screening, antibiotic prophylaxis, and improved postsurgical care. The implications of this study extend beyond Ethiopia, offering valuable insights for similar healthcare contexts in resource‐limited regions. Through a retrospective cross‐sectional analysis, this study seeks to shed light on the unique challenges and opportunities for improving surgical outcomes in the context of abdominal surgeries in Ethiopia.

## 2 Material and Methods

### 2.1 Study Setting and Period

The research was conducted at Bichena Primary Hospital, situated in Northwest Ethiopia, approximately 265 km away from Addis Ababa, the capital of Ethiopia. Bichena Primary Hospital is part of the Amhara regional state and falls under the administration of the Amhara Regional Health Bureau. It is located in Bichena Town within the East Gojjam Zone and was established in the Ethiopian calendar year 2007 (EC). The hospital serves a catchment population of 522,000, comprising 261,522 males and 260,478 females, attending to approximately 58,960 patients annually. The facility is equipped with 36 inpatient beds and conducts around 958 emergency abdominal operations within a 2‐year span. The hospital's workforce consists of 216 staff members, including 113 health professionals.

### 2.2 Study Design

An institution based retrospective cross‐sectional study was conducted.

### 2.3 Source Population

All patients who underwent surgery and were admitted to Bichena Primary Hospital.

### 2.4 Study Population

All patients who underwent open abdominal surgery and were admitted to Bichena Primary Hospital between September 1, 2022, and August 31, 2023.

### 2.5 Eligibility Criteria

This study included patients of all ages who underwent open abdominal surgeries, irrespective of the circumstances surrounding the surgery. However, certain exclusions were applied. Patients who underwent abdominal surgery at a different hospital and were subsequently referred to Bichena Primary Hospital were excluded from the study. Additionally, cases resulting in patient death within 3 days post‐surgery were excluded. Furthermore, patients with incomplete medical charts were also excluded from the analysis.

### 2.6 Sample Size and Sampling

The sample size for the study was determined utilizing the single population proportion formula, taking into account a proportion of surgical site infection (*P* = 12.3%) from a previous study [23], with a 95% confidence level, a 5% margin of error, and a 10% contingency. Based on these parameters, the estimated sample size was 183. All records of abdominal operations conducted at Bichena Primary Hospital between September 2019 and August 2020 were identified through the examination of operation room, surgical ward, and recovery log books. From these log books, a list of medical record numbers (MRN) pertaining to patients who underwent abdominal operations and were admitted to Bichena Primary Hospital during the retrieval period was compiled. Subsequently, a simple random sampling technique was applied to select the required sample of 183 patients for the study.

### 2.7 Data Collection Tool and Procedure

A checklist derived from the World Health Organization (WHO) Surgery Safety guidelines and the Nosocomial Infection National Surveillance Scheme (NINSS), developed collaboratively by NINSS and the Centers for Disease Control and Prevention (CDC) [26, 27], was adapted to align with the specific objectives and characteristics of this study and its setting. This modified tool was utilized for data extraction from the study participants.

The data collection process was carried out by two Bachelor of Science (BSc) nurses who underwent training specifically for this data collection task. A checklist, tailored for the purpose of this study, was prepared to guide the data collection process. Information encompassing socio‐demographic details, clinical factors, and pre‐ and intra‐operative data relevant to surgical site infection (recorded at the initiation of treatment) was retrieved from the patients' records.

### 2.8 Data Quality Control

Data quality was ensured through the utilization of a standardized checklist derived from the WHO Surgery Safety guidelines and the NINSS [26], which was tailored for the specific requirements of this study. Before the actual survey, the modified checklist underwent a pretest involving 5% of the study sample size. Adjustments were made to the checklist based on the insights gained from the pretest. The investigator provided daily supervision throughout the data collection process. Regular checks for completeness, accuracy, and consistency of the collected data were conducted by the investigator on a daily basis.

### 2.9 Variables in the Study

Socio‐demographic attributes such as age, sex, and residence, as well as clinical factors like the type of anesthesia administered (categorized as general or regional), the circumstance under which the surgery took place (emergency or elective), the utilization of prophylactic antibiotics, wound contamination classes at time of surgery (ranging from clean to dirty), the presence of additional comorbidities, malignancy, diabetes mellitus, immunosuppressant usage, associated medical illnesses, operative findings, the timing of the procedure, and the duration of hospital stay, were evaluated to determine their association with the dependent variable, which is the occurrence of surgical site infection.

### 2.10 Operational Definition

**Surgical site infection:** an infection that occurs after abdominal surgery on the site where the surgery took place and which is diagnosed by the clinician (yes/no).

**Abdominal Operation:** a surgical procedure that is done in the person's abdominal region to diagnose or treat a medical condition (CDC, 2018).

**Comorbidity:** any medical condition known during the time of surgery and has been written on the chart of the patients.

**Length of hospital stay:** In this study, the length of hospital stay was measured as the total number of days a patient spent in the hospital. To facilitate the analysis, the duration was categorized into four groups; 5–7 days, 8–14 days, 15–21 days, and ≥ 22 days. The cut‐off of 22 days was selected based on a natural distribution of the data, where most patients had shorter stays. This categorization allowed for analysis of the relationship between hospital stay duration and the risk of surgical site infections.

### 2.11 Data Processing and Analysis

The collected data underwent a thorough examination for completeness, identification of inconsistencies, and detection of missing values. Subsequently, the data were coded and input into Epi‐data version 4.6, then exported for analysis using SPSS version 25. Both bivariable and multivariable analyses were conducted to pinpoint factors associated with surgical site infections.

In the multivariate analysis, various established risk factors linked to the development of SSIs were assessed. Multivariate stepwise logistic regression analyses were employed to account for multiple predictive factors and their potential interactions. A significance level of 0.25 was set for entry into the model. The independence of these factors was characterized using multivariable χ2 and *p*‐values. The relationship between the outcomes of interest and each independent factor was quantified using odds ratios (OR) and 95% confidence intervals (95% CI). All tests were two‐sided, and significance was established at *p* < 0.05.

The multivariate goodness‐of‐fit was evaluated using the Hosmer‐Lemeshow test (0.762). Predictive validity was assessed by calculating the area under the receiver operating characteristics (AUROC) curve. The interpretation of accuracy based on the AUROC curve considered the model as poor if within 0.51 and 0.69, useful if within 0.70 and 0.79, and good if ≥ 0.80. The results were conveyed through narrative text, tables, and graphs.

### 2.12 Ethical Consideration

The ethical considerations of this study were reviewed and approved by the Ethical Committee of Bahir Dar University, College of Medicine and Health Sciences. Additionally, official permission to conduct the study was obtained from Bichena Primary Hospital. The purpose of the study was communicated to supportive staff, including card room workers and surgical staff, and informed, voluntary, written, and signed consent was obtained from the head of the hospital. Measures were put in place to ensure the confidentiality of patient information throughout the study process.

## 3 Results

### 3.1 Socio‐Demographic Characteristics of Study Participants

A total of 164 patient charts were scrutinized in the investigation, excluding 19 charts due to incomplete information. The mean age of the study participants was 28.4 years (standard deviation = ±8 years), ranging from 8 to 87 years. The majority of respondents (51.8%) fell within the age bracket of 25–64 years. Children under the age of 15 accounted for 11 (6.7%) individuals, while elders aged 65 and above constituted 32 (19.5%) of the participants. The median (IQR) length of stay was 11(7) days with the majority 78 (47%) of patients stayed less than 7 days. Among all patients, 99 (60.4%) were male, and the majority (73%) of the study participants hailed from rural areas. Out of all the individuals in the study, 120 patients (constituting 73.2%) experienced a waiting period exceeding 24 h for their surgery after being admitted to the hospital, as shown in Table 1.

**Table 1:** Socio‐demographic characteristics of patients who underwent abdominal operation in Bichena Primary Hospital, 2022.

Variables	Category	Frequency	Percent (%)
Age	≤ 14 years	11	6.7
	15–24 years	36	22.0
	25–64 years	85	51.8
	>= 65 years	32	19.5
Sex	Male	99	60.4
	Female	65	39.6
Residence	Rural	120	73.2
	Urban	44	26.8
Length of stay	5–7days	78	47.6
	8–14 days	56	34.1
	15–21 days	23	14.0
	≥ 22 days	8	4.9
Admission to operation time	< 24hrs	44	26.8
	≥ 24hrs	120	73.2

### 3.2 Comorbidity, Wound Class, and Preoperative Characteristics

In the entire cohort under investigation, 36 individuals (constituting 22% of the total participants) presented with a concurrent medical condition. Within this subgroup, HIV/AIDS was identified in 13 cases (36.2%), hypertension in 8 patients (22.2%), diabetic mellitus in 8 cases (22.2%), and anemia in 7 cases (19.4%). Out of all subjects included in the study, 19 individuals (11.6%) had undergone blood transfusion before surgery, and preoperative prophylactic antibiotics were administered to 142 patients (86.6%) before their operations. The majority of patients (84.8%) received general anesthesia, and iodine served as the primary antiseptic for most cases (56.7%). Additionally, the majority of patients (*n* = 145) experienced a blood loss of less than 1000 mL during the surgical procedure, as shown in Table 2.

**Table 2:** Comorbidity, wound class, and preoperative characteristics of patients who underwent abdominal surgery at Bichena Hospital, 2023.

Characteristics	Category	Frequency	Percentage (%)
Comorbidity	Yes	36	22.0
	No	128	78.0
Type of comorbidity (*n* = 36)	Hypertension	8	22.2
	HIV/AIDS	13	36.2
	Anemia	7	19.4
	Diabetic mellitus	8	22.2
Wound class at time of surgery	Clean	46	28.1
	Clean contaminated	38	23.2
	Contaminated	59	35.9
	Dirty	21	12.8
Preoperative blood transfusion	Yes	19	11.6
	No	145	88.4
Prophylactics antibiotics given	Yes	142	86.6
	No	22	13.4
Type of anesthesia used	General	139	84.7
	Spinal	25	15.3
The volume of blood loss	< 1000 ml	145	88.4
	≥ 1000 ml	19	11.6
Type of antiseptic used	Saline based	11	6.7
	Iodine based	93	56.7
	Saline and iodine	42	25.6
	Saline and alcohol	18	10.9

Abbreviations: AIDS= acquired immunodeficiency syndrome, HIV = human immunodeficiency virus.

### 3.3 Reason for Surgery and Case Related Characteristics

The preponderance of procedures conducted consisted of emergency abdominal operations, constituting 139 cases (84.8%). Within this category, 15 cases (10.8%) were categorized as traumatic, while 124 cases (89.2%) were deemed non‐traumatic. Specifically, in the overall pool of abdominally operated cases, acute appendicitis represented 42 cases (25.6%), peritonitis accounted for 27 cases (16.5%), large bowel obstruction for 17 cases (10.4%), small bowel obstruction for 14 cases (8.5%), perforated peptic ulcer disease for 14 cases (8.5%), compound bowel obstruction for 6 cases (3.7%), and trauma for 15 cases (9.1%). Of the total traumatic cases (*n* = 15), thoracic‐abdominal injury constituted 6 cases (40%), penetrating abdominal injury for 7 cases (46.6%), and blunt abdominal injury for 2 cases (13.3%), as presented in Table 3.

**Table 3:** Reason for surgery and case‐related characteristics of patients who underwent abdominal surgery in Bichena Hospital, 2023.

Characteristic	Category	Frequency	Percent (%)
Circumstance of surgery (*n* = 164)	Elective	25	15.2
	Emergency	139	84.8
Emergency case type (*n* = 139)	Traumatic	15	10.8
	Non traumatic	124	89.2
Indication for surgery (*n* = 164)	small bowel obstruction	14	8.5
	Intussusceptions	4	2.4
	large bowel obstruction	17	10.4
	Compound bowel obstruction	6	3.7
	Perforated PUD	14	8.5
	Acute appendicitis	42	25.6
	Peritonitis	27	16.5
	Trauma	15	9.2
	Other elective cases	25	15.2
Types of traumas (*n* = 15)	Penetrating abdominal injury	7	46.6
	Blunt abdominal injury	2	13.3
	Thoracic‐abdominal injury	6	40

Abbreviation: PUD= peptic ulcer disease.

### 3.4 Magnitude of Surgical Site Infection

Out of the 164 patients who underwent abdominal operations, 44 patients developed surgical site infections, resulting in an incidence rate of 26.8% (95% CI: 20.1%–33.5%). Among the observed cases of surgical site infections, superficial SSI accounted for 27 (16.5%) cases, followed by deep postoperative SSI with 14 (8.5%) cases, and organ space infections with 3 (1.8%) cases.

### 3.5 Factors Associated With Surgical Site Infection

In the bivariable analysis, factors associated with surgical site infection development (*p*‐value < 0.25) included patient age (*p* = 0.033), associated medical illness (*p* = 0.002), wound contamination class at surgery (*p* = 0.001), total hospital stay duration (*p* = 0.020), type of antiseptic used (*p* = 0.105), and estimated intraoperative blood loss volume (*p* = 0.116). However, in multivariable logistic regression, only associated medical illness and hospital stay length were significantly linked to surgical site infection. Patients with pre‐existing medical conditions were more than three times as likely to develop a surgical site infection (SSI) compared to those without such conditions (AOR = 3.37, 95% CI: [1.32, 8.60], *p* < 0.001). Additionally, the likelihood of developing SSI decreased significantly with shorter hospital stays. Specifically, patients who stayed for 5–7 days had a 91% lower chance of developing SSI (AOR = 0.09, 95% CI: [0.07, 0.95], *p* = 0.002). Similarly, those who stayed 8‐14 days had a 78% lower likelihood (AOR = 0.22, 95% CI: [0.08, 0.75], *p* = 0.005), and patients who stayed 15‐21 days had a 72% lower chance (AOR = 0.28, 95% CI: [0.05, 0.60], *p* = 0.001) of developing SSI compared to patients with postoperative stays longer than 22 days, as presented in Table 4.

**Table 4:** Multivariable logistic regression to identify factors associated with surgical site infection in Bichena Primary Hospital, northwestern Ethiopia, 2023.

Variables	Categories	Surgical site infection		COR (95% CI)	AOR (95% CI)
		Yes	No		
Age	0–14 years	6 (13.64%)	5 (4.20%)	1.59 (0.39,6.40)	0.59 (0.42,1.52)
	15–24 years	6 (13.64%)	30 (25.00%)	9.54 (0.65,9.85)	0.44 (0.08,2.34)
	25–64 years	11 (25.00%)	74 (61.60%)	2.84 (0.88,6.75)	0.07 (0.02,1.24)
	>= 65 years	21 (47.72%)	11 (9.20%)	Reff	Reff
Comorbidity	Yes	17 (38.64%)	19 (15.8%)	0.30 (0.14,0.65)	3.37 (1.32,8.59)*
	No	27 (61.36%)	101 (84.2%)	Reff	Reff
Type of antiseptic used	Saline based	5 (11.36%)	6 (5.00%)	1.04 (0.23,4.70)	2.22 (0.26,8.91)
	Iodine based	20 (45.46%)	73 (60.83%)	1.34 (0.12,1.98)	0.44 (0.10,1.91)
	Saline and iodine	11 (25.0%)	31 (25.84%)	0.44 (0.14,1.41)	0.76 (0.14,4.03)
	Saline and alcohol	8 (18.18%)	10 (8.33%)	Reff	Reff
Wound class at the time of surgery	Clean	6 (13.64%)	40 (33.33%)	Reff	Reff
	Clean contaminated	6 (13.64%)	32 (26.67%)	1.68 (0.99,2.85)	0.44 (0.07,2.57)
	Contaminated	22 (50.0%)	37 (30.83%)	5.33 (0.23,7.75)	0.58 (0.14,2.41)
	Dirty	10 (22.72%)	11 (9.17%)	6.67 (0.82,8.72)	0.32 (0.05,1.88)
volume of blood loss	< 1000 ml	36 (81.82%)	109 (90.8%)	0.45 (0.17,1.21)	0.45 (0.10,2.01)
	> 1000 ml	8 (18.18%)	11 (9.17%)	Reff	Reff
Total duration of hospital stays	5–7 days	6 (13.64%)	71 (59.16%)	7.10 (1.35,8.20)	0.09 (0.07,0.95)*
	8–14 days	21 (47.72%)	35 (29.17%)	1.33 (1.21,4.61)	0.22 (0.08,0.75)*
	15–21 days	14 (31.82%)	9 (7.50%)	0.38 (0.07,0.92)	0.28 (0.05,0.60)*
	≥ 22 days	3 (6.82%)	5 (4.17%)	Reff	Reff

* *p* value < 0.5.

Abbreviations: AOR = Adjusted odds ratio, CI = Confidence interval, COR = Crude odds ratio.

## 4 Discussion

This study aimed to estimate the magnitude of surgical site infection and its determinant factors among patients who underwent abdominal surgery in Bichena Hospital. The magnitude of SSI in this hospital was 26.8% (95% CI: 20.1%–33.5%). Prolonged hospital stays and having associated medical illness were found to be independent factors associated with SSI in this study.

This incidence is higher than rates reported in some studies but falls within the spectrum of SSI prevalence documented in other regions. The SSI incidence of 26.8% reported in this study is within the range observed in various healthcare settings globally and locally [11, 14, 28, 29, 30, 31]. This finding was Comparable to SSI rates reported in Asela Referral and Teaching Hospital (23.3%), Dessie Referral Hospital (23.4%), Jimma University Medical Center (21.1%), Hawassa University Teaching Hospital (24.6%) in Ethiopia [18, 19, 28]. It was also consistent with the study conducted in Tanzania (26%), Sudan (27.5%), Pakistan (29.8%), and Nepal (23%) when we compared to abroad [14, 29, 30, 31].

In comparison to global data, the prevalence of surgical site infections (SSIs) in Ethiopia aligns with rates seen in other low‐income settings, though it is generally higher than those observed in high‐income countries. Globally, the risk of SSIs varies significantly, with rates ranging from 2.5% to 30.9% in sub‐Saharan Africa [25, 26]. The 26.8% rate of SSI observed in this study is on the higher end of the spectrum, similar to findings from countries such as Tanzania, Sudan, and Pakistan [14, 29, 30, 31]. However, it is notable that this rate exceeds those observed in government hospitals and public healthcare settings in Ethiopia, where rates tend to be lower [12, 20, 22, 32]. Additionally, it was higher than the findings in South Western Uganda (16.4%), Ghana (16.2%), and global collaborative studies (9.3%) outside Ethiopia [2, 10, 33]. While this rate of SSI is relatively high compared to many studies, it remains lower than the findings reported in Bolan Medical College and Abbottabad tertiary care hospitals [34, 35]. This variation in SSI rates could be attributed to differences in study populations, healthcare infrastructure, adherence to infection control practices, and variations in surgical techniques.

The study identifies concurrent medical conditions as significant risk factors for the development of surgical site infections (SSIs). Patients with such conditions were more than three times as likely to experience SSI compared to those without these issues. This finding aligns with numerous studies that emphasize the role of comorbidities, such as hypertension, immunosuppression, and cancer, in compromising the host's defense mechanisms and increasing the risk of infection post‐surgery [15, 18, 35, 36]. Managing these conditions is particularly challenging in resource‐limited settings, where access to diagnostic tools, medications, and specialized care is often restricted. The need for early identification and management of these comorbidities is critical to minimizing their impact on patient outcomes and reducing SSI risk.

The study underscores the role of hospital length of stay as a crucial factor influencing the likelihood of SSI. Patients with shorter postoperative stays exhibited a substantially lower likelihood of developing SSI compared to those with longer stays. Similar findings have been reported in studies conducted in various healthcare settings [13, 20, 22]. While patients with longer stays exhibited a higher likelihood of developing SSI, it is important to acknowledge the potential for reverse causality, where SSI itself could contribute to prolonged hospital stays. Infections may lead to extended hospitalization due to the need for additional medical interventions and complications. Moreover, more invasive surgeries, which increase both the risk of SSI and the length of hospital stays, should be considered as a potential confounding factor. This bidirectional relationship between hospital stay duration and SSI risk, along with the influence of surgical invasiveness, suggests that future studies should explore these interactions further. Optimizing postoperative care and facilitating timely discharges could reduce both the incidence of SSI and the length of hospital stays, ultimately improving healthcare efficiency.

### 4.1 Strengths and Limitations of Study

The main outcome was derived from information collected through secondary data sources, but a significant number of values were missing. This may impact the accuracy of the research findings. Additionally, the data was retrospectively gathered through chart reviews, introducing limitations in ensuring data quality. Notably, there is a lack of published data on the prevalence of surgical site infections and related factors among patients who underwent abdominal operations in our country. The study revealed noncompliance with perioperative protocols, including issues like inadequate glove and dressing changes by surgeons. Violations of protocols in the surgical ward were noted due to a shortage of surgical materials, raising concerns about improper wound care and contamination. The study covered outpatient SSIs but acknowledged potential underreporting due to patients lost to follow‐up after outpatient surgery, highlighting uncertainty in post‐discharge surveillance accuracy and hindering precise SSI rate determination.

## 5 Conclusion

SSIs significantly burden hospitals serving large patient populations, particularly in resource‐limited settings. This study revealed a high SSI rate of 26.8% among abdominal surgery patients. The presence of associated medical illnesses and prolonged hospital stays was significantly linked to SSI occurrence. These findings emphasize the importance of improved management of comorbidities and timely discharge planning. Strengthening infection prevention protocols and targeting high‐risk patients may significantly reduce SSI incidence and improve surgical outcomes, particularly in resource‐limited healthcare settings.

## Author Contributions

**Tesfaye Shumet Mekonnen:** methodology, conceptualization, investigation, funding acquisition, resources, data curation, formal analysis, software, project administration, writing – review and editing, writing – original draft. **Shegaw Getinet:** conceptualization, data curation, formal analysis, funding acquisition, methodology, investigation, project administration, resources, software, writing – review and editing. **Amare Mebrat Delie:** methodology, visualization, writing – review and editing, software, supervision, project administration, resources. **Eneyew Talie Fenta:** methodology, investigation, project administration, validation, resources, supervision, software, writing – review and editing. **Fassikaw Kebede Bizuneh:** methodology, project administration, resources, writing – review and editing, software. **Wubetu Woyraw:** conceptualization, data curation, formal analysis, methodology, investigation, project administration, resources, software, writing – original draft.

## Ethics Statement

The ethical review board of Bahir Dar University ethical review board cleared this study to be conducted after checking compatibility with human subjects based on the Helsinki declaration with refill number (RCS, TT & UIL;2127/2023). In addition, the IRB board of Bahir Dar University has approved written informed consent from the hospital. Written informed consent was obtained from the Bichena primary hospital administration.

## Conflicts of Interest

The authors declare no conflicts of interest.

## Transparency Statement

The lead author Tesfaye Shumet Mekonnen affirms that this manuscript is an honest, accurate, and transparent account of the study being reported; that no important aspects of the study have been omitted; and that any discrepancies from the study as planned (and, if relevant, registered) have been explained.

## Data Availability

The data that support the findings of this study are available from the corresponding author upon reasonable request.

## References

[1] F. C. Brunicardi , D. K. Andersen , T. R. Billiar , et al., Schwartz's Principles of Surgery (McGraw‐Hill Education, 2019). 2, 11th Edition.

[2] A. Lubega , B. Joel , and N. Justina Lucy , “Incidence and Etiology of Surgical Site Infections Among Emergency Postoperative Patients in Mbarara Regional Referral Hospital, South Western Uganda,” Surgery Research and Practice 2017 (2017): 1–6. 10.1155/2017/6365172 PMC5266862 28168215

[3] S. Ogihara , T. Yamazaki , T. Maruyama , et al., “Prospective Multicenter Surveillance and Risk Factor Analysis of Deep Surgical Site Infection After Posterior Thoracic And/Or Lumbar Spinal Surgery in Adults,” Journal of Orthopaedic Science 20, no. 1 (2015): 71–77. 25366698 10.1007/s00776-014-0669-1

[4] B. Allegranzi , S. B. Nejad , C. Combescure , et al., “Burden of Endemic Health‐Care‐Associated Infection in Developing Countries: Systematic Review and Meta‐Analysis,” Lancet 377, no. 9761 (2011): 228–241. 21146207 10.1016/S0140-6736(10)61458-4

[5] R. S. Nair , R. R. Nair , S. Munshi , S. Walkay , and N. G. Radhamony , “Analysis of Postoperative Complications Following Elective Arthroscopic Surgeries of the Knee‐ a Retrospective Cohort Study,” Annals of Medicine & Surgery 77 (2022): 103546. 35638045 10.1016/j.amsu.2022.103546 PMC9142390

[6] S. Bibi , G. A. Channa , T. R. Siddiqui , and W. Ahmed , “Frequency and Risk Factors of Surgical Site Infections in General Surgery Ward of a Tertiary Care Hospital of Karachi, Pakistan,” International Journal of Infection Control 7, no. 3 (2011): 1, 10.3396/ijic.v7i3.6093.

[7] G. Collaborative and A. Bhangu Surgical Site Infection After Gastrointestinal Surgery in High‐income, Middle‐income, and Low‐income Countries. 10.1016/S1473-3099(18)30101-4 PMC5910057 29452941

[8] A. Bhangu , A. O. Ademuyiwa , M. L. Aguilera , et al., “Surgical Site Infection After Gastrointestinal Surgery in High‐Income, Middle‐Income, and Low‐Income Countries: A Prospective, International, Multicentre Cohort Study,” Lancet Infectious Diseases 18, no. 5 (2018): 516–525. 29452941 10.1016/S1473-3099(18)30101-4 PMC5910057

[9] C. Danwang , J. J. Bigna , J. N. Tochie , et al., “Global Incidence of Surgical Site Infection After Appendectomy: A Systematic Review and Meta‐Analysis,” BMJ Open 10, no. 2 (2020): e034266. 10.1136/bmjopen-2019-034266 PMC7045165 32075838

[10] G. Collaborative , “Determining the Worldwide Epidemiology of Surgical Site Infections After Gastrointestinal Resection Surgery: Protocol for a Multicentre, International, Prospective Cohort Study (Globalsurg 2),” BMJ Open 7, no. 7 (2017): e012150. 10.1136/bmjopen-2016-012150 PMC5577891 28733294

[11] R. Mezemir , A. Seid , T. Gishu , T. Demas , and A. Gize , “Prevalence and Root Causes of Surgical Site Infections at an Academic Trauma and Burn Center in Ethiopia: A Cross‐Sectional Study,” Patient Safety in Surgery 14 (2020): 3. 31921353 10.1186/s13037-019-0229-x PMC6945788

[12] B. B. Billoro , M. H. Nunemo , and S. E. Gelan , “Evaluation of Antimicrobial Prophylaxis use and Rate of Surgical Site Infection in Surgical Ward of Wachemo University Nigist Eleni Mohammed Memorial Hospital, Southern Ethiopia: Prospective Cohort Study,” BMC Infectious Diseases 19, no. 1 (2019): 298. 30940093 10.1186/s12879-019-3895-5 PMC6446332

[13] C. Degbey , A. Kpozehouen , D. Coulibaly , et al., “Prevalence and Factors Associated With Surgical Site Infections in the University Clinics of Traumatology and Urology of the National University Hospital Centre Hubert Koutoukou Maga in Cotonou,” Frontiers in Public Health 9 (2021): 629351. 33643993 10.3389/fpubh.2021.629351 PMC7902708

[14] R. S. E. E. Hassan , S. O. S. Osman , M. A. S. Aabdeen , W. E. A. Mohamed , R. S. E. E. Hassan , and S. O. O. Mohamed , “Incidence and Root Causes of Surgical Site Infections After Gastrointestinal Surgery at a Public Teaching Hospital in Sudan,” Patient Safety in Surgery 14, no. 1 (2020): 45. 33372624 10.1186/s13037-020-00272-4 PMC7722425

[15] A. Afenigus , T. Gualu , and A. Tadesse , “Surgical Site Infection and Associated Factors Among Adult Patients Admitted in West and East Gojjam Zone Hospitals, Amhara Region,” Ethiopia 6 (2020): 6.

[16] O. Ojo , B. Owolabi , A. Oseni , O. Kanu , and O. Bankole , “Surgical Site Infection in Posterior Spine Surgery,” Nigerian Journal of Clinical Practice 19, no. 6 (2016): 821–826. 27811458 10.4103/1119-3077.183237

[17] M. A. Cataldo , G. Granata , and N. Petrosillo , “Antibacterial Prophylaxis for Surgical Site Infection in the Elderly: Practical Application,” Drugs & Aging 34, no. 7 (2017): 489–498. 28589466 10.1007/s40266-017-0471-9

[18] D. Misganaw , B. Linger , and A. Abesha , “Surgical Antibiotic Prophylaxis Use and Surgical Site Infection Pattern in Dessie Referral Hospital, Dessie, Northeast of Ethiopia,” BioMed Research International 2020 (2020): 1695683. 32258103 10.1155/2020/1695683 PMC7104263

[19] G. Misha , L. Chelkeba , and T. Melaku , “Incidence, Risk Factors and Outcomes of Surgical Site Infections Among Patients Admitted to Jimma Medical Center, South West Ethiopia: Prospective Cohort Study,” Annals of Medicine & Surgery 65 (2021): 102247. 33898031 10.1016/j.amsu.2021.102247 PMC8058519

[20] K. Fisha , M. Azage , G. Mulat , and K. S. Tamirat , “The Prevalence and Root Causes of Surgical Site Infections in Public Versus Private Hospitals in Ethiopia: A Retrospective Observational Cohort Study,” Patient Safety in Surgery 13 (2019): 26. 31333761 10.1186/s13037-019-0206-4 PMC6617908

[21] W. Bank Ethiopia Overview. (2021). https://dataworldbankorg/country/ethiopia.

[22] N. Awoke , A. Arba , and A. Girma , “Magnitude of Surgical Site Infection and Its Associated Factors Among Patients Who Underwent a Surgical Procedure at Wolaita Sodo University Teaching and Referral Hospital, South Ethiopia,” PLoS One 14, no. 12 (2019): e0226140. 31805161 10.1371/journal.pone.0226140 PMC6894841

[23] W. S. Shiferaw , Y. A. Aynalem , T. Y. Akalu , and P. M. Petrucka , “Surgical Site Infection and Its Associated Factors in Ethiopia: A Systematic Review and Meta‐Analysis,” BMC Surgery 20, no. 1 (2020): 107. 32423397 10.1186/s12893-020-00764-1 PMC7236319

[24] J. O. Osakwe , G. A. Nnaji , R. C. Osakwe , U. Agu , and H. N. Chineke , “Role of Premorbid Status and Wound Related Factors in Surgical Site Infection in a Tertiary Hospital in Sub‐Saharan Africa,” Family Practice Reports 1, no. 1 (2014): 2.

[25] W. H. Organization Report On The Burden of Endemic Health Care‐associated Infection Worldwide. (2016). https://wwwwhoint/gpsc/country_work/burden_hcai/en/.

[26] E. M. Cooke , R. Coello , J. Sedgwick , et al., “A National Surveillance Scheme for Hospital Associated Infections in England,” Journal of Hospital Infection 46, no. 1 (2000): 1–3. 11023716 10.1053/jhin.2000.0801

[27] K. Nishiwaki and T. Ichikawa , “[WHO Surgical Safety Checklist and Guideline for Safe Surgery 2009],” Masui The Japanese Journal Of Anesthesiology 63, no. 3 (2014): 246–254. 24724433

[28] B. Deribe , W. Jemebere , and G. Bekele Surgical Site Infection Prevalence and Associated Factors in Hawassa University Comprehensive Specialized Hospital, Southern Ethiopia 2019.

[29] S. Giri , B. P. Kandel , S. Pant , P. J. Lakhey , Y. P. Singh , and P. Vaidya , “Risk Factors for Surgical Site Infections in Abdominal Surgery: A Study in Nepal,” Surgical Infections 14, no. 3 (2013): 313–318. 23672239 10.1089/sur.2012.108

[30] F. U. Khan , Y. Fang , Z. Khan , et al., “Occurrence, Associated Risk Factors, and Treatment of Surgical Site Infections in Pakistan,” European Journal of Inflammation 18 (2020): 2058739220960547.

[31] B. Mawalla , S. E. Mshana , P. L. Chalya , C. Imirzalioglu , and W. Mahalu , “Predictors of Surgical Site Infections Among Patients Undergoing Major Surgery at Bugando Medical Centre In Northwestern Tanzania,” BMC Surgery 11 (2011): 21. 21880145 10.1186/1471-2482-11-21 PMC3175437

[32] T. Legesse Laloto , D. Hiko Gemeda , and S. H. Abdella , “Incidence and Predictors of Surgical Site Infection in Ethiopia: Prospective Cohort,” BMC Infectious Diseases 17, no. 1 (2017): 119. 28158998 10.1186/s12879-016-2167-x PMC5290629

[33] A. A. A. Bediako‐Bowan , K. Mølbak , J. A. L. Kurtzhals , E. Owusu , S. Debrah , and M. J. Newman , “Risk Factors for Surgical Site Infections in Abdominal Surgeries in Ghana: Emphasis on the Impact of Operating Rooms Door Openings,” Epidemiology and Infection 148 (2020): e147. 32605670 10.1017/S0950268820001454 PMC7398855

[34] F. Sattar , Z. Sattar , M. Zaman , and S. Akbar , “Frequency of Post‐Operative Surgical Site Infections in a Tertiary Care Hospital in Abbottabad, Pakistan,” Cureus 11, no. 3 (2019): e4243. 31131166 10.7759/cureus.4243 PMC6516612

[35] L. Shah , S. Baber , S. Lehri , M. Khan , and I. Baloch , “Surgical Site Infections, an Experience at Tertiary Care Hospital,” Journal of University Medical & Dental College 12, no. 3 (2021): 185–192.

[36] A. Shakir , D. Abate , F. Tebeje , and F. Weledegebreal , “Magnitude of Surgical Site Infections, Bacterial Etiologies, Associated Factors and Antimicrobial Susceptibility Patterns of Isolates Among Post‐Operative Patients in Harari Region Public Hospitals, Harar, Eastern Ethiopia,” Infection and Drug Resistance 14 (2021): 4629–4639. 34764659 10.2147/IDR.S329721 PMC8577515

